# Hope and Creative Self-Efficacy as Sequential Mediators in the Relationship Between Family Socioeconomic Status and Creativity

**DOI:** 10.3389/fpsyg.2020.00438

**Published:** 2020-03-17

**Authors:** Yinyinzi Yang, Xiaobo Xu, Wenling Liu, Weiguo Pang

**Affiliations:** ^1^School of Psychology and Cognitive Science, East China Normal University, Shanghai, China; ^2^Institute of General Education, Shanghai Academy of Educational Sciences, Shanghai, China

**Keywords:** socioeconomic status, hope, creative self-efficacy, creative ideation, creativity

## Abstract

The purpose of this study was to evaluate how socioeconomic status (SES) predicts individual creativity through the mediating roles of hope and creative self-efficacy (CSE). Participants were recruited from ten universities in Mainland China. Students’ SES, hope, CSE, and creativity were assessed via the socioeconomic status scale, the adult hope scale, the creative self-efficacy scale, and the Runco Ideational Behavior Scale. Correlational analyses indicate that SES, creative ideation, hope, and CSE were significantly and positively associated with each other. Path analyses revealed that hope and CSE played sequential mediating roles in the link between SES and creative ideation. These findings suggest that hope and CSE underlie the effect of SES on individuals’ creative ideation.

## Introduction

Creativity is defined as the ability to generate original and useful ideas or solutions to problems (Sternberg and Lubart, 1999). As an important human ability, creativity vita to the arts, technology, and science and it is affected by numerous factors (Simonton, 2004; Weisberg, 2006; Tillander, 2011). According to the Investment theory of Sternberg and Lubart (1992), creativity is co-influenced by intelligence, knowledge, personality, thinking style, motivation, and many environmental variables, among which family socioeconomic status (SES) is a variable worthy of in-depth investigation.

A number of relational studies have established a positive link between SES and creativity (Kaltsounis, 1974; Daugherty and White, 2008; Dai et al., 2012). However, the mechanism by which SES influences creativity is not fully understood. For instance, although previous research has illustrated the mediating roles of intelligence (Shi and Shen, 2007), motivation (Dai et al., 2012) and personality (Zhang et al., 2018), fewer studies focused on the mediating effect of the expectational factors (e.g., hope). In his 30-year longitudinal study, Torrance (2004) found that personal perceptions of the future are far more predictive of later creative achievement than past achievements or traits. Moran (2010) suggested that individuals’ future perceptions toward creativity (e.g., the hopes and risks of being creative in the future) can affect their future creative achievement. Thus, the main purpose of this study was to explore the possible effect of hope on the association of SES and creativity.

The second purpose of this study is to investigate the roles of creative self-efficacy (CSE) in the association of SES and creativity. This is because previous research has documented that hope is strongly influence self-efficacy (Sezgin and Erdogan, 2015).

Many studies have found a creativity gap between individuals from low and high SES families (Mitchell, 1975; Shi and Shen, 2007). Investment theory (Sternberg and Lubart, 1992) suggests that creativity is influenced by cognitive as well as environmental factors. The environment is shaped by family SES, which encompasses parental education, parental occupation, and family income (Duncan et al., 1972). SES has been associated with different facets of creativity, such as everyday creativity, social creativity, and creative ideation (Richards et al., 1988; Dai et al., 2012; Zhang et al., 2018). For instance, research has shown high SES background students have higher levels of creativity than do low SES background students (Dai et al., 2012).

Many conditions associated with low SES are related to creativity. Low SES students have fewer resources, such as books, electronic products, and opportunities to travel, which limits their knowledge-related background (Brooks-Gunn and Duncan, 1997; Evans, 2004). This, in turn, reduces knowledge activation in creative idea generation tasks (Rietzschel et al., 2007). Meanwhile, diminished access to resources also leads to adverse cognitive, financial, and emotional states (Kraus et al., 2012). Consequently, when faced with unmet needs and external threats or problems, those from low SES families find it difficult to be creative (Collins and Amabile, 1999).

Socioeconomic status as a contextual factor can also have a positive relationship with creativity self-efficacy (Karwowski, 2011), a well-known predictor of creativity that is typically defined as the confidence one has in his/her ability to handle problems that require creative thinking and functioning (Barron and Harrington, 1981). For example, Beghetto (2006) claims that SES is one of the key factors which are related to middle and secondary school students’ CSE. Additionally, Karwowski (2004) found that parental education level creates a variety of micro milieus in the home, therefore positively exerting and influence on their children’s CSE.

Hope refers to goal-directed thinking and consists of two elements, the motivation to achieve desired goals (agency) and the pathways to goal achievement (pathway) (Snyder et al., 1991, 1997; Snyder, 2000). Empirically, studies have revealed that hope is positively associated with SES and creativity (Snyder, 2002; Kraus et al., 2012; Rego et al., 2012).

Snyder (2002) asserts that low SES individuals tend to have lower hope compared to high SES individuals. Specifically, contextual factors constrain low SES individuals by restricting their goals, knowledge, and social connections (Bradley and Corwyn, 2002; Kraus et al., 2012) and making it difficult for them to find viable pathways to achieve their goals. Additionally, these stressful contexts increase an individual’s focus on external forces that cannot be controlled, thus reducing internal motivation to complete tasks (Dixson et al., 2017). To summarize, low SES reduces hope through the constraint of resources on viable pathways and through the reduction of attention to goals (Snyder, 2000).

Recently, some studies have found that hope predicts a series of positive outcomes, including academic achievement (Dixson et al., 2017), well-being (Guse and Vermaak, 2011), and creativity (Rego et al., 2012). The influence of hope on creativity can be illustrated by the creative dual-process model (Baas et al., 2013). According to this model, approach-traits can enhance creativity through cognitive flexibility and avoidance-traits can enhance creativity through cognitive persistence. In the present study, we propose that hope has the capacity to function as a type of approach-trait and thus increases creativity through cognitive flexibility. Specifically, based on the definition of hope, individuals with higher levels of hope are not only good at finding viable pathways but also generating more alternative pathways to reach their goals. Accordingly, high hope individuals can be more flexible, allowing for greater creativity. For example, Rego et al. (2014) found that most hopeful individuals seek creative ways to pursue their goals. Further, when they face difficulties, they seek creative ways to overcome obstacles (Luthans et al., 2007). Additionally, compared to those with low levels of hope, individuals with higher hope have greater agency and are more willing to invest in goal-directed efforts (Snyder, 2002). For instance, previous research indicates that hopeful employees enjoy pursuing their goals (Oldham and Cummings, 1996). As a result, they are more intrinsically motivated and prefer to implement their agency in creative ways.

Hope has also been found to increase self-efficacy (Avey et al., 2008). As such, CSE may mediate the relationship between hope and creativity. In the creative domain, CSE is an example of self-efficacy, which originates from four sources (Bandura, 1977, 1986): experience with solving problems, watching familiar individuals cope, encouragement, and emotional and physical motivation. Hope affects each of these four sources of self-efficacy. More specifically, high hope individuals have more successful experiences, because they have more chances to try, and they are more willing to face challenges (Snyder, 2000). Also, as mentioned before, high SES individuals have both high hope and abundant social resources, giving them examples of success and encouragement, thus leading to higher levels of CSE. Finally, high hope individuals are highly motivated to find viable pathways to accomplish tasks and achieve goals (Snyder, 2002; Shalley and Gilson, 2004). Thus, this greater agency also can generate higher CSE when coping with creative problems.

A number of research has indicated that CSE can increase creativity by enhancing perceptions of self-competence and promoting interests in engagement in creative activities (Beghetto and Karwowski, 2017; Puente-Diaz and Cavazos-Arroyo, 2018). Accordingly, many previous studies have identified CSE as an important predictor of different forms of creativity (Beghetto et al., 2011; Jaiswal and Dhar, 2016). For example, CSE has a stronger predictive effect than any other individual or context predictors of creativity (Hammond et al., 2011). Furthermore, CSE also can mediate the relationship between many environmental and personal factors and creativity. For example, Puente-Díaz et al. (2019) found that CSE mediated the association between multicultural experiences and creative potential.

The current study explores the sequential mediation model of SES, hope, CSE, and creativity by assessing creative ideation to represent creativity. Creative ideation, a cognitive ability to generate many creative ideas, is a pivotal part of everyday creativity and eminent creativity. It is also an important aspect of creativity, referred to as creative potential, that is often measured by creativity tests (e.g., Alternate Uses Test) as well as self-report questionnaires (Runco Ideation Behavior Scale) (Runco et al., 2001; Plucker et al., 2006).

The literature review suggests the following hypothesis.

*Hypothesis 1*: SES, creativity, hope, and CSE are positively related to each other.

*Hypothesis 2*: SES has an indirect effect on creativity as mediated by hope.

*Hypothesis 3*: SES has an indirect effect on creativity as mediated by CSE.

*Hypothesis 4*: SES has an indirect effect on creativity as mediated by hope and then CSE.

The purpose of this study is to illustrate the route from SES to creativity through hope and CSE. The mediation roles of hope and CSE are important because if hope, and CSE, do partially mediate the relationship between SES and creativity, they might provide possible ways to diminish low SES’s negative impact on creativity. In other words, effective hope or CSE interventions might improve the creativity for low SES individuals (Kraus et al., 2012).

## Materials and Methods

### Participants and Procedure

To test our hypotheses, we surveyed 1003 undergraduate students from 10 different universities which are located in five different provinces of Mainland China. The students spent roughly 15 min completing the survey during breaks between their classes. We excluded the cases for which the data were missing, reducing the number of participants to 607 (females = 378). Some data were missing for 396 participants, mainly due to participants’ null response to family income (*N* = 329), a somewhat sensitive item in SES research field (Hoff et al., 2002; Karwowski, 2011). All the students were undergraduates (freshman = 49.3%, sophomore = 34.9%, junior = 11.5%, senior = 1.8%, fifth-year = 2.5%). Ages ranged from 18 to 26 years (*M* = 20.24 years, *SD* = 1.28 years). The students’ majors included art, engineering, education, management, and medicine. None of the students had answered these questionnaires previously and whose participation in the survey was voluntary, and respondents were compensated for completing it.

This study was carried out in accordance with the recommendations of the ethics committee of East China Normal University with written informed consent from all subjects in accordance with the Declaration of Helsinki. The protocol was approved by the ethics committee of East China Normal University.

### Measurements

#### Family Social and Economic Status

Socioeconomic status was measured using the Duncan Socioeconomic Index (SEI) (Stevens and Featherman, 1981). SEI includes three key factors; parental education, parental occupation, and family income. First, we assigned respondents one of five possible scores for parental education (below elementary school and elementary school = 1; junior high school = 2, senior high school = 3; bachelor’s degree = 4; postgraduate and doctoral degrees = 5). Second, following Nan and Bian (1991), parental occupation was divided into five major groups: farming (= 1), manufacturing (= 2), transportation (= 3), service and office work (= 4), administrative and professional (= 5). Third, we added the scores of the father and mother for parental education and for parental occupation. Fourth, family income was measured as the l total family monthly income. Fifth, we standardized the scores. Finally, a principal component analysis was conducted to check whether the three SES-factors could be seen as one single factor. The PCA and the screeplot gave support for a one-factor solution. Then according to the procedure presented by previous researchers (Vyas and Kumaranayake, 2006; Krishnan, 2010; Heshmat et al., 2016) the factor loadings of the three standardized factors was used to weight their respective contribution to the combined SES-variable (SES = Zincome * 0.265 + Zeducation * 0.491 + Zoccupation * 0.496).

#### Hope

The adult hope scale (AHS) consists of 12 items along three dimensions (Snyder et al., 1991). Two main dimensions include four items for hope-agency (HA) and four items for hope-pathway (HP). The last dimension is a filler. Data for the AHS were self-reported and rated using a 5-point Likert scale, which ranged from 1 (*definitely false*) to 5 (*definitely true*). Examples of HA and HP were as follows: “My past experiences have prepared me well for my future” (HA); “I can think of many ways to get the things in life that are most important to me” (HP). The internal reliability of HA and HP were 0.68 and 0.73, respectively. The total scale of AHS showed good reliability with α = 0.82.

#### Creative Self-Efficacy

Creative self-efficacy was measured using a subscale of the Short Scale of Creative Self (SSCS) consisting of six items (Karwowski et al., 2013). An example of an item in the CSE scale is: “I am sure I can deal with problems requiring creative thinking.” All the items were self-rated using a 5-point Likert scale, which ranged from 1 (*definitely not*) to 5 *(definitely yes*). The CSE scale demonstrated good internal reliability (α = 0.73).

#### Creative Ideation

Creative ideation was assessed using the Runco Ideational Behavior Scale (RIBS) (Runco et al., 2001). It contains 23 items that were rated on a 5-point Likert-type scale from 1(*never*) *to* 5 (*very often*). An example of an item from this scale is “I have ideas about new inventions or about how to improve things.” It showed good internal reliability (α = 0.85 for the total scale).

#### Control Variable

Gender is considered to be an important control variable (Liu et al., 2017) and as such was included in data analyses (female was coded as 0, male was coded as 1).

#### Analytic Strategy

All data were analyzed using SPSS 24. First, descriptive analyses were conducted with the variables of interest for the total sample. Then, the Pearson’s correlations between variables were calculated to provide a preliminary test of the *Hypotheses 1*. Next, serial mediation analysis was conducted using PROCESS 3.3 macro (Model 6) for SPSS (Hayes, 2017) to test *Hypotheses 2, Hypotheses 3, and Hypotheses 4*. SES was entered as the predictor. Hope and CSE were entered as mediators. Gender was entered as covariate. The mediation analyses were conducted for creative ideation. We used 5000 boot-strapping resamples to generate a 95% percentile confidence interval (CI) for the indirect effects we estimated. If the CI of the indirect effect does not include zero, the null hypothesis is rejected.

## Results

### Common Method Variance Test

We used Harman’s single factor analysis to test the common method variance. The results indicated that the first factor explained only 31.48% (lower than 40%) of the total variance. Therefore, common method bias was unlikely to be a concern in this study.

### Preliminary Analyses

The results of descriptive statistics and correlations are presented in Table 1. As predicted, the score for SES was significantly and positively correlated with the total score of RIBS. Further, the score of hope was positively correlated with SES and RIBS individually. In addition, CSE was positively associated with SES, hope, and RIBS.

**TABLE 1:** Means, standard deviations, and correlations of all variables (*N* = 607).

**Variables**	**M**	**SD**	**SES**	**AHS**	**CSE**
SES	0.00	1.00	–	–	–
AHS	28.43	4.62	0.13**	–	–
CSE	3.27	0.72	0.12**	0.55**	–
RIBS	67.66	10.48	0.14**	0.33**	0.43**

SES, social and economical statues; AHS, the adult hope scale; CSE, creative self-efficacy, subscale of short scale of creative scale; RIBS, the Runco Ideational Behavior Scale; **p < 0.01.

### Test of Mediation

**FIGURE 1:**
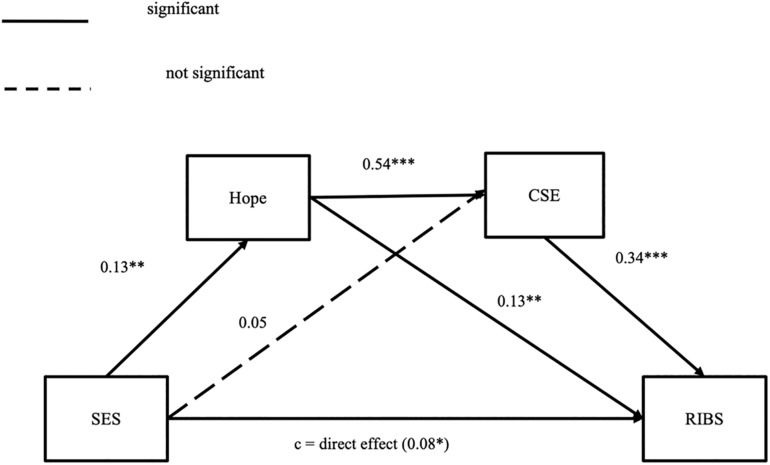
Sequential mediation model regarding the mediating effects of hope and creative self-efficacy on the relation between SES and creativity. All the path coefficients were standardized. *N* = 607. ***p < 0.001; **p < 0.01; *p < 0.05.

We used the Hayes macro PROCESS (Hayes, 2017) to explore the sequential mediation relationship. Hope and CSE were entered as mediators between SES and creativity. Gender was controlled for as a potential confounding factor in the mediation analysis. We conducted serial mediation analysis to test the mediating role of hope and CSE between the SES and creative ideation. The results are presented in Figure 1 and Table 2.

**TABLE 2:** Indirect effects and confidence intervals of meditational analyses, controlling for gender.

			**95% CI**	
**Model pathways**	**Effect value**	**SE**	**Lower**	**Upper**
SES→HOPE→RIBS	0.17^a^	0.10	0.02	0.40
SES→CSE→RIBS	0.17	0.12	−0.05	0.41
SES→HOPE→CSE→RIBS	0.24^a^	0.09	0.09	0.44

*SES, social and economical statues; AHS, the Adult Hope Scale; CSE, creative self-efficacy, subscale of short scale of creative scale; RIBS, the Runco Ideational Behavior Scale; RIBS, the Runco Ideational Behavior Scale; SE, standard error. CI, confidence interval. ^*a*^Empirical 95% confidence interval does not overlap with zero.*

The total effect of SES on RIBS was 1.40 (95% CI: 0.58, 2.23). The direct effect of SES on creative ideation was positive and significant (direct effect = 0.82, 95% CI: 0.07, 1.57). The indirect effect of SES on creative ideation through hope was significant (indirect effect = 0.17, 95% CI = 0.02, 0.40). The indirect effect of SES on creative ideation through CSE was not significant (indirect effect = 0.17, 95% CI = −0.05, 0.41). There was a significant positive indirect effect of SES on creative ideation (RIBS score) through hope and then CSE (indirect effect = 0.24, 95% CI: 0.09, 0.44). These results indicate that hope and CSE partially mediate the relationship between SES and creative ideation. In addition, the results of pairwise contrast of three indirect effects showed no significant difference.

## Discussion

The purpose of the current study was to explore the relationship between SES and creativity through the mediators of hope and CSE. First, the descriptive results are consistent with previous research that suggests that these variables are positively related (*Hypothesis 1*) (Tierney and Farmer, 2002; Avey et al., 2008; Dai et al., 2012; Kraus et al., 2012). Further, the mediation analysis revealed that hope partially mediates the effect of SES on creative ideation (*Hypothesis 2*); Hope and CSE partially mediate the relationship between SES and creative ideation (*Hypothesis 4*).

The results support *Hypothesis 1*, that SES significantly and positively relates to creativity, reconfirming previous research demonstrating that individuals with high SES have high levels of creativity (Dai et al., 2012). The results also indicate that high SES individuals who have ample resources tend to possess high levels of hope. These findings support previous research showing that individuals with different levels of SES perceive different levels of hope in similar situations. Low SES individuals are limited by context factors; therefore, they may not be able to collect enough information to develop an effective goal toward the future (Dixson et al., 2017). SES also shows a significant and positive link with CSE, thus supporting the view that higher SES is beneficial to the development of CSE (Beghetto, 2006; Karwowski, 2011). Additionally, these results also suggest that hope significantly and positively relates to CSE, confirming the positive relationship between hope and self-efficacy that has been found in previous literature (Sezgin and Erdogan, 2015) and extending this relationship to the creative domain. According to the definition of hope, Avey et al. (2008) interpreted self-efficacy as the conviction in ones’ ability to generate multiple pathways, take actions and ultimately succeed in goal attainment. Hence, based on our results, CSE can be interpreted as the conviction in one’s ability to (a) generate multiple creative pathways, (b) take actions toward creative problem solving, and (c) ultimately be successful in creative problem-solving. As hypothesized, CSE also shows a significant and positive link with creativity. It supports the previous findings on the relationship between creativity and CSE (Beghetto et al., 2011; Richter et al., 2012). For instance, Farmer and Tierney (2017) revealed that a series of studies has reported the positive link between CSE and creativity relates outcomes. It suggests that the confidence in creative problem-solving will influence the creative performance (Karwowski, 2011; Puente-Diaz and Cavazos-Arroyo, 2016).

Additionally, the results from the correlation analyses indicate a significant positive link between hope and creativity (*Hypothesis 1*). The mediation analysis revealed a significant direct effect of hope on creativity and mediation roles of hope between SES and creativity (*Hypothesis 2*). The mediation analysis helps to explain the processes of how SES affects creativity. SES promote individual’s creativity because those individuals in high SES develop greater hope, thus being more creative. Unlike previous research (Shi and Shen, 2007; Zhang et al., 2018) which focused on the mediating roles of past achievement or personal traits (e.g., intelligence, personality), this study explored the mechanisms involved in how SES shapes creativity from the perspective of personal perceptions toward the future. Specifically, SES dictates what resources individuals have had access to in the past (I have the necessary resources to be creative). As we emphasized previously, how those resources are used to create a better future is a significant issue in the creativity research domain, and the indirect effect of SES on creativity suggest that hope play an important role in the facilitation of creativity. Kraus et al. (2012) have found that high SES individuals with abundant resources perceive themselves in more agentic ways. They tend to focus on their own internal state, goals. Similarly, high SES individuals with abundant resources can broaden their thinking pathways and agentic thoughts in the pursuit of their goals (Lopez et al., 2000b). Moreover, these pathways and agentic thoughts are two key elements of hope (Snyder, 2002). In addition, Moran (2010) has suggested that creativity can benefit from hope. High hope individuals may abound in flexible thinking and creativity because they believe they can follow more alternative pathways toward the future (Lopez et al., 2000a).

Notably, the indirect effect of SES on creativity through CSE was not significant in this sequential mediation model (*Hypothesis 3)*. Similarly, the direct effect of SES on CSE was also not significant in this integrative model, although the correlation between these two variables was significant (*r* = 0.12). To our knowledge, these results may suggest that hope can play a fully mediating roles in the relationship between SES and CSE. In another words, high SES individuals may foster greater hope to develop a higher level of CSE. This result expands the findings of Karwowski (2011)’s research regarding the relationship between SES and CSE by taking a new possible mediator (hope) into account. Accordingly, the indirect effect of SES on creativity through CSE may be diminished when taking hope into account in the sequential mediation model. Our findings also suggest that there is an indirect effect of hope on creativity through CSE. Previous research indicates that employees’ hope as psychological capital can predict their creativity (Rego et al., 2014). Based on our results, a possible explanation of this result may be that hope affects employees’ creativity by increasing their CSE.

Furthermore, the sequential mediation analyses result supports our *Hypothesis 4*. It takes a new important mediator (CSE) into account to explain the mechanism by which SES affects creativity. The findings suggest that SES promote creativity because individuals in high SES develop greater hope, and this greater hope promote their CSE, thus being more creative. The *Hypothesis 2* has suggested that hope is an important mediator in the relationship between SES and creativity. Further, researchers have illustrated that high hope individuals have positive perceptions of their competence to solve future problems creatively (Snyder et al., 1997). Similarly, Michael (2000) has suggested that increased hope can promote self-efficacy’s role in future behavioral changes. In other words, hope can increase individuals’ CSE in future creative performance. In addition, Tierney and Farmer (2011) have found that changes in CSE will lead to corresponding changes in creative performance in a 6-month longitudinal study. Taken together, this sequential mediation path provides a clear description of the process by which SES affects creativity through hope and then CSE.

Some studies have explored the associations between SES and creativity. However, the mechanism between these two variables has not been fully elucidated and our study is the first study of how hope and CSE mediate this relationship. We conducted this study in ten Chinese universities and were able to generate a large sample. This large sample size increases our confidence in the external validity of the results.

However, this study has some limitations that suggest directions for future research. Firstly, RIBS is a self-report survey, and response bias is inevitable in this type of method. Future researchers should use other experimental methods to collect data on creativity, including the alternative use test, Remote Associates Test and Torrance Tests of Creative Thinking. Secondly, the participants were college students in China, which may limit our ability to generalize the results across other age groups and cultures. Lastly, the cross-sectional design does not allow causal inferences. Future experimental or longitudinal designs are needed to confirm our results.

The findings of this study have some theoretical and practical implications. Our results indicate that SES affects creativity through hope and CSE, both of which play a significant role in creative ideation. These findings indicate that creativity might be developed through interventions targeted toward SES levels. Hope intervention may help individuals from low SES backgrounds improve their creativity directly or by increasing their CSE. CSE showed great influence on creativity, indicating that increasing confidence may enhance low SES individuals’ creativity.

## Data Availability Statement

The raw data supporting the conclusions of this article will be made available by the authors, without undue reservation, to any qualified researcher.

## Ethics Statement

The studies involving human participants were reviewed and approved by the Ethics Committee of East China Normal University. The patients/participants provided their written informed consent to participate in this study.

## Author Contributions

YY contributed the initial idea generation, analyzed/interpreted the data and wrote this manuscript. XX revised this manuscript substantially. WL was responsible for the data collection. WP made contributions to supervision, initial idea, study design improvement, interpretation of the results, and some critical reviews of this manuscript.

## Conflict of Interest

The authors declare that the research was conducted in the absence of any commercial or financial relationships that could be construed as a potential conflict of interest.

## References

[B1] Avey J. B. Wernsing T. S. Luthans F. (2008). Can positive employees help positive organizational change? impact of psychological capital and emotions on relevant attitudes and behaviors. *J. Appl. Behav. Sci.* 44 48–70. 10.1177/0021886307311470

[B2] Baas M. Roskes M. Sligte D. Nijstad B. A. De Dreu C. K. (2013). Personality and creativity: the dual pathway to creativity model and a research agenda. *Soc. Pers. Psychol. Compass* 7 732–748. 10.1111/spc3.12062

[B3] Bandura A. (1977). Self-efficacy: toward a unifying theory of behavioral change. *Psychol. Rev.* 84 191–215. 10.1037/0033-295x.84.2.191 847061

[B4] Bandura A. (1986). The explanatory and predictive scope of self-efficacy theory. *J. Soc. Clin. Psychol.* 4 359–373.

[B5] Barron F. Harrington D. M. (1981). Creativity, intelligence, and personality. *Annu. Rev. Psychol.* 32 439–476.

[B6] Beghetto R. A. (2006). Creative self-efficacy: Correlates in middle and secondary students. *Creat. Res. J.* 18 447–457.

[B7] Beghetto R. A. Karwowski M. (2017). “Toward untangling creative self-beliefs,” in *The Creative Self*, eds Karwowski M. Kaufman J. C. (Amsterdam: Elsevier), 3–22. 10.1016/b978-0-12-809790-8.00001-7

[B8] Beghetto R. A. Kaufman J. C. Baxter J. (2011). Answering the unexpected questions: exploring the relationship between students’ creative self-efficacy and teacher ratings of creativity. *Psychol. Aesthet. Creat. Arts* 5 342–349. 10.1037/a0022834

[B9] Bradley R. H. Corwyn R. F. (2002). Socioeconomic status and child development. *Annu. Rev. Psychol.* 53 371–399. 11752490 10.1146/annurev.psych.53.100901.135233

[B10] Brooks-Gunn J. Duncan G. J. (1997). The effects of poverty on children. *Future Child* 7 55–71. 9299837

[B11] Collins M. A. Amabile T. M. (1999). I5 motivation and creativity. *Handb. Creat.* 297 1051–1057.

[B12] Dai D. Y. Tan X. Marathe D. Valtcheva A. Pruzek R. M. Shen J. (2012). Influences of social and educational environments on creativity during adolescence: does SES matter? *Creat. Res. J.* 24 191–199. 10.1080/10400419.2012.677338

[B13] Daugherty M. White C. S. (2008). Relationships among private speech and creativity in head start and low—socioeconomic status preschool children. *Gift. Child Q.* 52 30–39. 10.1177/0016986207311059

[B14] Dixson D. D. Keltner D. Worrell F. C. Mello Z. (2017). The magic of hope: hope mediates the relationship between socioeconomic status and academic achievement. *J. Educ. Res.* 111 507–515. 10.1080/00220671.2017.1302915

[B15] Duncan O. D. Featherman D. L. Duncan B. (1972). *(Socioeconomic) Background and Achievement.* Seminar Press: ıCambridgeı, MA

[B16] Evans G. W. (2004). The environment of childhood poverty. *Am. Psychol.* 59 77–92. 14992634 10.1037/0003-066X.59.2.77

[B17] Farmer S. M. Tierney P. (2017). “Considering creative self-efficacy: its current state and ideas for future inquiry,” in *The Creative Self*, eds Karwowski M. Kaufman J. C. (Amsterdam Elsevier), 23–47. 10.1016/b978-0-12-809790-8.00002-9

[B18] Guse T. Vermaak Y. (2011). Hope, psychosocial well-being and socioeconomic status among a group of South African adolescents. *J. Psychol. Afr.* 21 527–533. 10.1080/14330237.2011.10820493

[B19] Hammond M. M. Neff N. L. Farr J. L. Schwall A. R. Zhao X. (2011). Predictors of individual-level innovation at work: a meta-analysis. *Psychol. Aesthet. Creat. Arts* 5 90–105. 10.1037/a0015978 19702361

[B20] Hayes A.F. (2017). *Introduction to Mediation, Moderation, and Conditional Process Analysis: A Regression-Based Approach.* New York, NY: Guilford Publications.

[B21] Heshmat R. Qorbani M. Ghoreshi B. Djalalinia S. Tabatabaie O. R. Safiri S. (2016). Association of socioeconomic status with psychiatric problems and violent behaviours in a nationally representative sample of Iranian children and adolescents: the CASPIAN-IV study. *BMJ Open* 6:e011615. 10.1136/bmjopen-2016-011615 27531729 PMC5013516

[B22] Hoff E. Laursen B. Tardif T. Bornstein M. (2002). Socioeconomic status and parenting. *Handb. Parent.* 8 231–252.

[B23] Jaiswal N. K. Dhar R. L. (2016). Fostering employee creativity through transformational leadership: Moderating role of creative self-efficacy. *Creat. Res. J.* 28 367–371. 10.1080/10400419.2016.1195631

[B24] Kaltsounis B. (1974). Race, socioeconomic status and creativity. *Psychol. Rep.* 35 164–166. 10.2466/pr0.1974.35.1.164 4429626

[B25] Karwowski M. (2004). Dzieci twórcze czy konformistyczne? Wartosci wychowawcze Polaków 1992–2002.[Creative or conformist children? Poles socialization values 1992–2002]. *Ruch Pedagogiczny* 5 23–43.

[B26] Karwowski M. (2011). It doesn’t hurt to ask(But sometimes it hurts to believe: polish students’ creative self-efficacy and its predictors. *Psychol. Aesthet. Creat. Arts* 5 154–164. 10.1037/a0021427

[B27] Karwowski M. Lebuda I. Wisniewska E. Gralewski J. (2013). Big five personality traits as the predictors of creative self-efficacy and creative personal identity: does gender matter? *J. Creat. Behav.* 47 215–232. 10.1002/jocb.32

[B28] Kraus M. W. Piff P. K. Mendoza-Denton R. Rheinschmidt M. L. Keltner D. (2012). Social class, solipsism, and contextualism: how the rich are different from the poor. *Psychol. Rev.* 119 546–572. 10.1037/a0028756 22775498

[B29] Krishnan V. (2010). *Constructing an Area-Based Socioeconomic Index: A Principal Components Analysis Approach.* Edmonton: Early Child Development Mapping Project.

[B30] Liu W. Pan Y. Luo X. Wang L. Pang W. (2017). Active procrastination and creative ideation: the mediating role of creative self-efficacy. *Pers. Individ. Diff.* 119 227–229. 10.1016/j.paid.2017.07.033

[B31] Lopez S. J. Floyd R. K. Ulven J. C. Snyder C. R. (2000a). “Hope therapy: helping clients build a house of hope,” in *Handbook of Hope: Theory, Measures, and Applications*, ed. Snyder C. R. (Cambridge, MA: Academic Press).123–150.

[B32] Lopez S. J. Gariglietti K. P. McDermott D. Sherwin E. D. Floyd R. K. Rand K. (2000b). “Hope for the evolution of diversity: on leveling the field of dreams,” in *Handbook of Hope*, ed. Snyder C. R. (Amsterdam Elsevier), 223–242.

[B33] Luthans F. Youssef C. M. Avolio B. J. (2007). *Psychological Capital: Developing the Human Competitive Edge.* Oxford: Oxford University Press.

[B34] Michael S. T. (2000). “Hope conquers fear: Overcoming anxiety and panic attacks,” in *Handbook of Hope*, ed Snyder C. R. (Amsterdam: Elsevier), 301–319. 10.1016/b978-012654050-5/50018-x

[B35] Mitchell B. (1975). *Creativity and the Poverty Child.* Available online at: https://files.eric.ed.gov/fulltext/ED129037.pdf#page=51 (accessed September 25, 2019).

[B36] Moran S. (2010). “The roles of creativity in society,” in *The Cambridge Handbook of Creativity*, eds Kaufman J. C. Bernardino S. Sternberg R. J. ( Cambridge: Cambridge University Press), 74–90 10.1017/cbo9780511763205.006

[B37] Nan L. Bian Y. (1991). Getting ahead in urban China. *Am. J. Sociol.* 97 657–688. 10.4066/AMJ.2011.584 23390460 PMC3562959

[B38] Oldham G. R. Cummings A. (1996). Employee creativity: personal and contextual factors at work. *Acad. Manag. J.* 39 607–634. 10.3390/ijerph17031038 32041278 PMC7037383

[B39] Plucker J. A. Runco M. A. Lim W. (2006). Predicting ideational behavior from divergent thinking and discretionary time on task. *Creat. Res. J.* 18 55–63. 10.1207/s15326934crj1801_7

[B40] Puente-Diaz R. Cavazos-Arroyo J. (2016). An exploration of some antecedents and consequences of creative self-efficacy: The role of achievement goals, enjoyment and divergent thinking. *Creat. Theor. Res. Appl.* 3 19–33. 10.1515/ctra-2016-0002

[B41] Puente-Diaz R. Cavazos-Arroyo J. (2018). An exploration of some antecedents and consequences of creative self-efficacy among college students. *J. Creat. Behav.* 52 256–266. 10.1002/jocb.149

[B42] Puente-Díaz R. Toptas S. Arroyo J. Wimschneider C. Brem A. (2019). Creative potential and multicultural experiences: the mediating role of creative self-Efficacy. *J. Creat. Behav.* 10.1002/jocb.408

[B43] Rego A. Sousa F. Marques C. Cunha M. P. (2014). Hope and positive affect mediating the authentic leadership and creativity relationship. *J. Bus. Res.* 67 200–210. 10.1016/j.jbusres.2012.10.003

[B44] Rego A. Sousa F. Marques C. Cunha M. P. E. (2012). Retail employees’ self-efficacy and hope predicting their positive affect and creativity. *Eur. J. Work Organ. Psychol.* 21 923–945. 10.1080/1359432x.2011.610891

[B45] Richards R. Kinney D. K. Benet M. Merzel A. P. (1988). Assessing everyday creativity: characteristics of the lifetime creativity scales and validation with three large samples. *J. Pers. Soc. Psychol.* 54 476–485. 10.1037/0022-3514.54.3.476

[B46] Richter A. W. Hirst G. Van Knippenberg D. Baer M. (2012). Creative self-efficacy and individual creativity in team contexts: cross-level interactions with team informational resources. *J. Appl. Psychol.* 97 1282–1290. 10.1037/a0029359 22800186

[B47] Rietzschel E. F. Nijstad B. A. Stroebe W. (2007). Relative accessibility of domain knowledge and creativity: the effects of knowledge activation on the quantity and originality of generated ideas. *J. Exp. Soc. Psychol.* 43 933–946. 10.1016/j.jesp.2006.10.014

[B48] Runco M. A. Plucker J. A. Lim W. J. C. R. J. (2001). Development and psychometric integrity of a measure of ideational behavior. *Creat. Res. J.* 13 393–400. 10.1207/s15326934crj1334_16

[B49] Sezgin F. Erdogan O. (2015). Academic optimism, hope and zest for work as predictors of teacher self-efficacy and perceived success. *Educ. Sci. Theory Pract.* 15 7–19.

[B50] Shalley C. E. Gilson L. L. (2004). What leaders need to know: a review of social and contextual factors that can foster or hinder creativity. *Leadersh. Q.* 15 33–53. 10.1016/j.leaqua.2003.12.004

[B51] Shi B. G. Shen J. L. (2007). The relationships among family SES, intelligence, intrinsic motivation and creativity. *Psychol. Dev. Educ.* 23 30–34.

[B52] Simonton D. K. (2004). *Creativity in Science: Chance, Logic, Genius, and Zeitgeist.* Cambridge, MA: Cambridge University Press.

[B53] Snyder C. R. (2000). *Handbook of Hope: Theory, Measures, and Applications.* Cambridge, MA: Academic press.

[B54] Snyder C. R. (2002). Hope theory: rainbows in the mind. *Psychol. Inq.* 13 249–275. 10.1207/s15327965pli1304_01

[B55] Snyder C. R. Harris C. Anderson J. R. Holleran S. A. Irving L. M. Sigmon S. T. (1991). The will and the ways: development and validation of an individual-differences measure of hope. *J. Pers. Soc. Psychol.* 60 570–585. 10.1037/0022-3514.60.4.570 2037968

[B56] Snyder C. R. Hoza B. Pelham W. E. Rapoff M. Ware L. Danovsky M. (1997). The development and validation of the children’s hope scale. *J. Pediatr. Psychol.* 22 399–421. 9212556 10.1093/jpepsy/22.3.399

[B57] Sternberg R. J. Lubart T. I. (1992). Buy low and sell high: an investment approach to creativity. *Curr. Dir. Psychol. Sci.* 1 1–5. 10.1111/1467-8721.ep10767737

[B58] Sternberg R. J. Lubart T. I. (1999). The concept of creativity: prospects and paradigms. *Handb. Creat.* 1 3–15. 10.1017/cbo9780511807916.003

[B59] Stevens G. Featherman D. L. (1981). A revised socioeconomic index of occupational status. *Soc. Sci. Res.* 10 364–395. 10.1016/0049-089x(81)90011-9

[B60] Tierney P. Farmer S. M. (2002). Creative self-efficacy: its potential antecedents and relationship to creative performance. *Acad. Manag. J.* 45 1137–1148. 10.5465/3069429

[B61] Tierney P. Farmer S. M. (2011). Creative self-efficacy development and creative performance over time. *J. Appl. Psychol.* 96 277–293. 10.1037/a0020952 20954756

[B62] Tillander M. (2011). Creativity, technology, art, and pedagogical practices. *Art Educ.* 64 40–46. 10.1080/10401330902791248 19330692

[B63] Torrance E. P. (2004). Great expectations: creative achievements of the sociometric stars in a 30-year study. *J. Second. Gift. Educ.* 16 5–13. 10.4219/jsge-2004-465

[B64] Vyas S. Kumaranayake L. (2006). Constructing socio-economic status indices: how to use principal components analysis. *Health Policy Plann.* 21 459–468. 10.1093/heapol/czl029 17030551

[B65] Weisberg R. W. (2006). *Creativity: Understanding Innovation in Problem Solving, Science, Invention, and the Arts.* Hoboken, NJ: John Wiley & Sons.

[B66] Zhang D. Zhou Z. Gu C. Lei Y. Fan C. (2018). Family socio-economic status and parent-child relationships are associated with the social creativity of elementary school children: the mediating role of personality traits. *J. Child Fam. Stud.* 27 2999–3007. 10.1007/s10826-018-1130-4

